# Wharton’s Jelly Hydrogel: An Innovative Artificial Ovary for Xenotransplantation of Isolated Human Ovarian Follicles

**DOI:** 10.3390/biology14101340

**Published:** 2025-10-01

**Authors:** Farnaz Tajbakhsh, Somayeh Tavana, Mohammad Kazemi Ashtiani, Ashraf Moini, Christiani Andrade Amorim, Rouhollah Fathi

**Affiliations:** 1 Department of Developmental Biology, University of Science and Culture, Tehran 1461968151, Iran; tajbakhsh_farnaz@yahoo.com; 2 Department of Embryology, Reproductive Biomedicine Research Center, Royan Institute for Reproductive Biomedicine, ACECR, Tehran 1665659911, Iran; tavana62s@yahoo.com; 3 Department of Cell Engineering, Cell Science Research Center, Royan Institute for Stem Cell Biology and Technology, ACECR, Tehran 1665659911, Iran; mkajuv@yahoo.com; 4 Department of Endocrinology and Female Infertility, Reproductive Biomedicine Research Center, Royan Institute for Reproductive Biomedicine, ACECR, Tehran 1665659911, Iran; ashraf.moieni@gmail.com; 5 Breast Disease Research Center (BDRC), Tehran University of Medical Science, Tehran 1417935840, Iran; 6 Department of Gynecology and Obstetrics, Arash Women’s Hospital, Tehran University of Medical Sciences, Tehran 1653915981, Iran; 7 Pôle de Recherche en Gynécologie, Institut de Recherche Expérimentale et Clinique, Université Catholique de Louvain, B-1348 Brussels, Belgium; christiani.amorim@uclouvain.be

**Keywords:** decellularized Wharton’s jelly, artificial ovary, human ovarian follicle isolation, tissue engineering, xenotransplantation, alginate hydrogel

## Abstract

Background: An artificial ovary has emerged as a novel alternative approach to prevent the reintroduction of cancerous cells after ovarian tissue autotransplantation. This study evaluates the ability of decellularized Wharton’s jelly (dWJ) to facilitate human ovarian follicle growth in a xenotransplantation model. Materials and Methods: Two transplanted groups were established; one consisted of a decellularized Wharton’s jelly/alginate (dWJ/Alg) composite, and an alginate (Alg) group was used as the control group. Each artificial ovary received approximately 20 partially isolated viable human ovarian follicles, subsequently undergoing xenotransplantation into ovariectomized, non-immunodeficient NMRI mice. Grafts were extracted at 1, 2, 4, or 5 weeks for comprehensive histological and immunohistochemical evaluations. Additionally, mouse blood serum was collected for hormonal analysis. Results: H&E staining confirmed granulosa cell proliferation and follicle growth in dWJ/Alg after 1 week of grafting. While human ovarian-like structures and cell proliferation were visible in other grafts, follicles were not observed. Conversely, immunohistochemical staining for Vimentin, Ki67, and CD45 confirmed the presence of human cells, proliferative cells, and inflammatory cells, respectively. However, hormonal assays revealed no significant difference in estrogen or progesterone levels between the experimental groups. Conclusions: It seems that Wharton’s jelly/alginate hydrogel can be used as an artificial niche for simulating the ovarian environment, effectively supporting the growth of xenotransplants of isolated human follicles.

## Simple Summary

An artificial ovary is a new approach to help women and prepubertal girls with no time to delay their cancer treatment. This bioengineered scaffold can function as a supportive environment for the growth and development of follicles, and reduce the risk of malignant cell returning. Wharton’s jelly is a rich natural extracellular matrix obtained from the umbilical cord. In this study, Wharton’s jelly was combined with alginate to form an artificial ovary in order to observe the survival and growth of human ovarian follicles after xenotransplantation. Alginate hydrogel was used as a control group. All grafts were removed at 1, 2, 4, or 5 weeks for comprehensive histological staining, immunohistochemical evaluations, and hormonal analysis. Based on histological data, granulosa cell proliferation and follicle growth could be seen in Wharton’s jelly/alginate after 1 week of grafting. Human ovarian-like structures and cell proliferation could be seen in all grafts, although there were no follicles. In addition, immunohistochemical staining for Vimentin, Ki67, and CD45 confirmed the presence of human cells, proliferative cells, and inflammatory cells, respectively. However, there were no significant differences in estrogen or progesterone levels between the experimental groups. Generally, Wharton’s jelly/alginate hydrogel can effectively support the growth of xenotransplants of isolated human follicles.

## 1. Introduction

In recent decades, the remarkable improvement in cancer patient recovery rates and life expectancy [1,2] has prompted the exploration of fertility preservation methods, considering the potential adverse effects of cancer treatments. Based on factors such as age, medical condition, and marital status, options such as embryo or mature oocyte cryopreservation can offer the prospect of future pregnancies. Ovarian tissue cryopreservation (OTC) is considered the best option for prepubertal girls and women with late-stage cancers requiring immediate treatment [3]. OTC preserves ovarian cortex strips for later transplantation into the individual following cancer treatment [4].

Despite over 200 reported live births following OTC [5], there is a risk of reintroducing malignant cells in some conditions, such as leukemia, autoimmune disease, and Hodgkin’s, limiting its applicability [6,7]. Two alternative approaches aim to help these patients. The first involves in vitro culture, employing a two-step strategy of activating primordial follicles in the ovarian cortex, followed by cultivating the individual growing follicles for in vitro fertilization [8,9]. Another technique is the construction of an artificial ovary [8,10], which offers advantages over in vitro culture, including transplantation of follicles in a natural body environment within a three-dimensional structure, reestablishing endocrine function, and reducing the risk of malignant cell contamination [11,12].

An artificial ovary serves as a bioengineered scaffold that functions as a supportive environment for the growth and development of follicles and cells [13]. It offers advantages such as porosity and biodegradability, which facilitate the growth and migration of follicles and cells, as well as cell attachment and angiogenic ability [14,15]. Several animal experiments investigating the building of artificial ovaries (AOs) have utilized various materials, including polyethylene glycol (PEG) [16] as a synthetic scaffold, as well as natural hydrogels such as plasma clots [17,18], collagen [19], alginate [20,21], gelatin [22], or human or porcine decellularized ovary [23,24,25]. However, research into human ovarian tissue bioengineering is still limited due to restricted access to human ovarian tissue for research reasons and xenotransplantation problems. In human research, several materials such as plasma clots [26,27], fibrin [6,28], decellularized ovarian tissue [29], and PEGylated fibrin [30] have been employed as an ovarian matrix. The main goal of these studies is to assess the survival rate of follicles following xenotransplantation, both in the short term and in the long term.

ECM is made up of collagens; glycosaminoglycans; proteoglycans; and growth factors such as transforming growth factor (TGF), fibroblast growth factor (FGF), and vascular endothelial growth factor (VEGF) [12]. Ovarian follicles and cells are located in this matrix [12]. There are potential options for ovarian tissue bioengineering when using the decellularized extracellular matrix (dECM) or dECM-derived hydrogels [31,32]. Wharton’s jelly (WJ) is a rich source of collagen, proteoglycan, glycosaminoglycan, and different types of growth factors such as insulin-like growth factor-1 (IGF1) and platelet-derived growth factor (PDGF). It is derived from the umbilical cord of preterm infants [33] and has been explored in various studies [34,35].

Therefore, this study investigates the potential of WJ to function as an artificial ovary (AO), promoting the survival and growth of human ovarian follicles following transplantation. In the present investigation, we utilized a hydrogel made of dWJ and alginate (dWJ/Alg) to provide a suitable environment for isolated human ovarian follicles. Afterward, the produced structure was transplanted into mice for both short and long durations.

## 2. Materials and Methods

### 2.1. Ethics

The Research Ethics Committees of Royan Institute Academic Center for Education, Culture and Research (IR.ACECR.ROYAN.REC.1400.023 ethical ID; Date: 15 May 2021) approved the utilization of umbilical cords and human ovarian tissue samples. All samples, including umbilical cords and human ovarian tissues, were acquired with the explicit consent of the individuals, who signed informed consent forms.

### 2.2. Experimental Design

**Figure 1:**
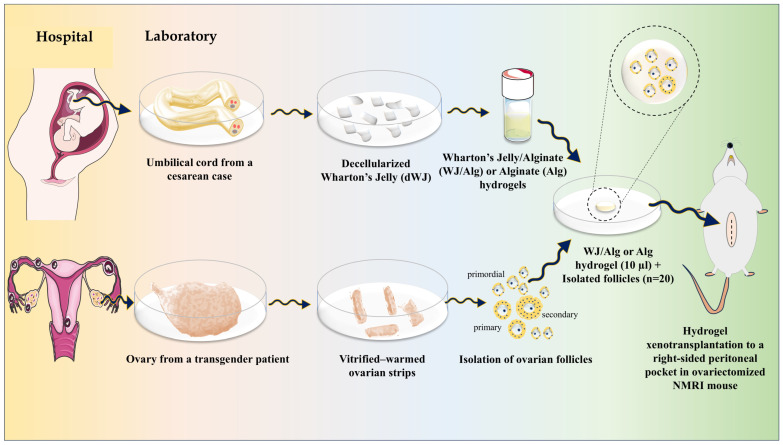
Graphical abstract of the construction of a human artificial ovary based on WJ/Alg.

This study was divided into two parts: artificial ovary construction and artificial ovary xenotransplantation. The study design is shown schematically in Figure 1.

In summary, Wharton’s jelly (WJ) was derived from the umbilical cord and underwent a decellularization process. Then, WJ solution was prepared and composed with alginate 1.5% to create a final dWJ/Alg solution (with a concentration of 1%). Next, primordial, primary, and preantral follicles (30–150 µm) were isolated from vitrified–warmed ovarian strips. After evaluating the viability of isolated follicles, approximately 20 partially isolated follicles were inserted into 10 µL of dWJ/Alg solution as the experimental group or alginate (Alg) solution alone as the control group. After adding a CaCl_2_ bath, the final artificial ovaries (hydrogels containing follicles) were prepared. Subsequently, every artificial ovary was transplanted into a peritoneal pocket in the right side of the abdomen of ovariectomized NMRI mice. The grafts were extracted at different time intervals of 1, 2, 4, or 5 weeks to conduct histological and immunohistochemical evaluations. Additionally, blood serum was collected for hormonal analysis. There were three replicates for each group.

### 2.3. WJ ECM Hydrogel

The method for decellularization of the WJ was conducted according to our earlier study [36], with some modifications as described in Table 1. In summary, umbilical cords (UCs) were obtained from 10 healthy women (aged between 25–35) who underwent cesarean surgery. Subsequently, each UC was transferred to the laboratory of the Royan Institute, where it was placed in a flask containing a cold (4 °C) physiological serum medium. After cleansing and extracting the endothelium components, Wharton’s jelly was divided into small fragments and underwent decellularization by incubation in 10 mM Tris (Sigma, St. Louis, MO, USA) and 0.1% ethylenediaminetetraacetic acid (EDTA; Sigma) (pH = 8) for 16 h at 4 °C. Then, they were placed into a new flask containing 10 mM base Tris-buffered saline (TBS; Tris–base 2.4 g/100 mL, NaCl 8.8 g/100 mL, HCl 12N 1.3 mL), 0.03% sodium dodecyl sulfate (SDS; Sigma), and 0.1% EDTA (pH = 7.6) on a shaker for 24 h. In the next step, after a wash with Tris, WJ strips were transferred into a new flask containing 50 mM Tris-HCl (Sigma) and 10 mM MgCl_2_ (pH = 7.5; Sigma) for 3 h on a shaker. In the final step, at least 7 washes were performed over 72 h with phosphate-buffered saline (PBS). A single rinse with PBS containing DNase (5 µL/mL; Sinaclon, Iran) and RNase (10 µL/mL; Sinaclon, Tehran, Iran) was performed for dWJ for 3–4 h.

### 2.4. Characterization of the Decellularized WJ

The decellularization process was verified by histological analysis (hematoxylin and eosin (H&E)), 4′,6-diamidino-2-phenylindole (DAPI) staining, and DNA content assessment.

The native and decellularized WJ were fixed in a 4% paraformaldehyde solution for at least 24 h. Afterward, blocks of paraffin were prepared. Random sections with a thickness of 5 µm were utilized for H&E staining. DAPI staining was employed to detect possible residual nuclei. In addition, the DNA content was assessed to evaluate the amount of remaining DNA in decellularized and native tissue. Therefore, 500 µL of digestion buffer and 20 µL of proteinase K were added to 10 mg of each sample. After vortexing, the mixture was incubated at 55 °C for 12 h. Subsequently, 500 µL of phenol–chloroform was added, followed by a 1-min vortex, and then left to stand for 20 min at room temperature (RT). The sample was centrifuged at 13,700 rpm for 20 min at 4 °C. After the appearance of three layers in solution, the uppermost layer was carefully collected and transferred to a new microtube. In the next step, the microtube was shaken, and an equal volume of cold ethanol (100%) was added. The mixture was then centrifuged at 8500 rpm for 15 min at 4 °C. The supernatant was removed, and 500 µL of cold ethanol 70% was added, followed by centrifugation at 8500 rpm for 15 min at 4 °C. The supernatant was removed again, and the sample was left to dry. Finally, 20 µL of H_2_O was added, and the optical density (OD) of each sample was measured using a nanodrop system (Thermo Fisher, Waltham, MA, USA). This entire process was conducted in triplicate for each method.

### 2.5. Production of dWJ/Alg and Alg Hydrogels

To initiate the process, 30 mg of dWJ powder was thoroughly mixed with 1 mg of pepsin and 1 mL of 1 N acetic acid for 24–48 h. Then, the prepared solution was neutralized with 500 µL of 0.5 N NaOH (Sigma). After that, 500 µL of the dWJ solution was combined with 500 µL of 1.5% alginate solution in a 1:1 ratio to prepare 1% dWJ/Alg solution. The final solution was sterilized under UV conditions and transferred into sterile vials for subsequent use.

### 2.6. Vitrification of Human Ovarian Tissue

Human ovary vitrification at the Royan Human Ovarian Tissue Bank (Royan OTB, Tehran, Iran) [37]. Human ovarian tissue (HOT) was obtained from fifteen transgender individuals (between 22 and 27 years of age) who underwent gender-affirming surgery. They were cut, vitrified in two steps, and stored in liquid nitrogen for future research [37].

### 2.7. Isolation of Human Ovarian Follicles

Two to four strips of vitrified ovarian tissues were warmed following the Royan OTB protocol [37]. Then, strips were chopped into 0.5 × 0.5 mm pieces with a tissue chopper (McIlwain Tissue Chopper, Cambridge, UK) and transferred into a Falcon tube containing HTCM, collagenase IA (1 mg/mL; Gibco, Grand Island, NY, USA), and 50 µL/mL Neutral Red (+NR group) (NR; Sigma, Kanagawa, Japan). The control group (without using NR), entitled the NR group, was chosen to compare the effect of NR on the time reduction of follicular detection and isolation.

The suspension underwent enzymatic digestion in a water bath at 37 °C for 60 min, with gentle agitation occurring every 20 min. Then, the enzyme was inactivated with cold HTCM and 20% human serum albumin (HSA). Insulin syringes were then used to isolate human ovarian follicles mechanically. Isolated ovarian follicles were transferred into new droplets using a 130 µm Pasteur pipet.

### 2.8. Evaluation of Follicular Viability

The survival of isolated follicles was assessed using a live/dead assay. For that purpose, random follicles were placed in drops of 100 µL PBS medium containing 2 µL calcein-AM (Invitrogen, Carlsbad, CA, USA) and 5 µL ethidium homodimer I (Invitrogen) and incubated for 30 min at 37 °C. Then, based on Chiti et al. [6], the viability of follicles was examined using fluorescent microscopy (Olympus BX51, Tokyo, Japan) and classified into four groups (V1–V4): (V1) viable follicles with no damage in oocyte or granulosa cells; (V2) follicles presenting 10% damaged cells; (V3) follicles with 10–50% dead cells; and (V4) follicles with more than 50% dead cells. V3 and V4 follicles were considered non-viable and consequently excluded from the study.

### 2.9. Artificial Ovary Xenotransplantation

Approximately 20 viable human follicles were placed in 10 µL of either Alg or dWJ/Alg solution and then soaked in a 50 µL CaCl_2_ bath to facilitate the formation of hydrogels, creating artificial ovaries (AOs). In the next step, twenty-four female mice (6–8 weeks) were kept under controlled conditions with a 12 h light/12 h dark cycle at 20–22 °C. Three biological replications were performed for each group, including the dWJ/Alg and Alg groups, in different weeks after transplantation (1, 2, 4, and 5 weeks). In addition, we had three ovariectomized mice for blood serum hormonal analysis. During the xenotransplantation procedure, mice were anesthetized using ketamine and xylazine based on their body weight, and the surgical process was performed under sterile conditions. 

To prepare for the xenotransplantation of artificial ovaries, the abdominal region of the mouse was cleaned and shaved, and then a 1.5 cm incision was made. After mouse ovariectomy, the AO structure was xenografted into the right side of the intraperitoneal pocket (n = 3 for each AO) and fixed with thin, non-absorbable sutures, and the abdominal wall and skin were closed using 6/0 sutures (Teb Keyhan, Karaj, Iran). Xenotransplanted AOs were retrieved at 7, 14, 28, and 35 days post-xenografting. To remove grafts, mice were anesthetized as described for the grafting procedure. After blood collection for hormonal analysis, the grafted AOs were detected and removed from the body.

### 2.10. Histological and Immunohistochemistry (IHC) Analyses

We fixed the grafted samples (n = 3 for each group) in 4% paraformaldehyde. After tissue processing, the prepared paraffin blocks were serially sectioned at 5 µm thickness for H&E staining. Smitz and Cortvrindt [38] classified follicles into different groups as follows: primordial follicles were characterized by a single flattened layer of granulosa cells (GCs) surrounding the oocyte; primary follicles were identified by a single layer of cuboidal GCs (30–60 µm); and secondary follicles were marked by two or more layers of cuboidal GCs (80–150 µm).

The fifth section, selected from a series of five sequential sections, was mounted on a positively charged slide for subsequent immunostaining. Immunohistochemistry analyses were performed with rabbit anti-CD45 antibody [EP322Y] (ab40763), rabbit anti-Vimentin antibody [EPR3776] (cytoskeleton) (ab92547), and rabbit anti-human Ki-67 monoclonal antibody [Clone SP6] (MAD-000310QD-3). Briefly, 4 μm thick paraffin sections were placed on poly-L-lysine-coated slides. Inhibition of endogenous peroxidase was achieved by incubating the sections with 10% hydrogen peroxidase in PBS for 10 min after they were deparaffinized. For heat antigen retrieval, the tissue sections were heated at 95 °C with sodium citrate buffer (10 mM, pH = 6) for 30 min. After washing with PBS, sections were incubated with primary antibodies for 50 min at RT. The slides were washed two times in PBS and immersed with secondary antibodies (MAD-000237Q) for 45 min at room temperature (RT). After washing the slides two times in PBS, the sections were incubated with the chromogen 3,3′-diaminobenzidine (DAB) for 10 min at RT and washed in tap water. Mayer’s hematoxylin dye was used to stain the nuclei. The slides were mounted and examined with a light microscope. Vimentin, a cytoplasmic protein, was used as a marker to identify human cells, especially mesenchymal-origin cells, which are most abundant in the ovarian stroma and GCs within growing follicles. Ki67, a nuclear antigen, was utilized to detect proliferating cells. CD45, an inflammatory marker, was used to detect inflammatory cells, such as leukocytes, macrophages, and T lymphocytes, thereby confirming the presence of immune responses against the xenograft.

### 2.11. Hormonal Analysis

Hormonal analysis was performed on mouse blood serum using the electrochemiluminescent immunoassay (ECLIA) method. Blood samples from xenografted mice were taken from the heart and incubated for 60 min at 4 °C. Then, the samples were centrifuged at 4000 rpm for 20 min, separating the uppermost medium containing the blood serum for subsequent hormonal analysis, focusing on estrogen and progesterone.

### 2.12. Statistical Analysis

Statistical analysis was performed using Prism software (version 9.0, GraphPad Software, San Diego, CA, USA), and the results are presented as the mean ± SD. The Shapiro–Wilk method was utilized to test the normality of the data. The parametric data underwent a one-way ANOVA and a *t*-test to compare between groups. Statistical significance was defined by a *p* value less than 0.05, which is indicated by a star (*). In addition, the symbols **, ***, and **** are employed to denote higher degrees of significance, with *p* < 0.01, *p* < 0.001, and *p* < 0.0001, respectively.

## 3. Results

### 3.1. Assessment of Decellularized WJ

**Figure 2:**
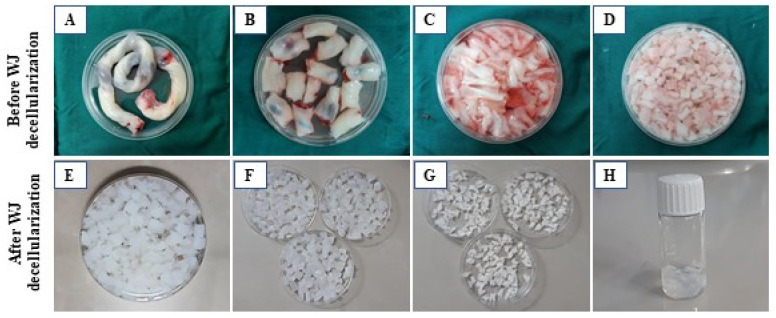
WJ processing overview. (**A**) Human umbilical cord (UC); (**B**) small UC fragments (10 × 5 × 1 mm^3^); (**C**) removal of vessel endothelial tissues for decellularization; (**D**) preparation of WJ pieces; (**E**,**F**) resulting decellularized WJ; (**G**) lyophilized WJ; (**H**) WJ hydrogel preparation.

The decellularization technique was conducted according to our previous investigation, with some modifications. The information is briefly shown in Table 1 and Figure 2A–H.

**Figure 3:**
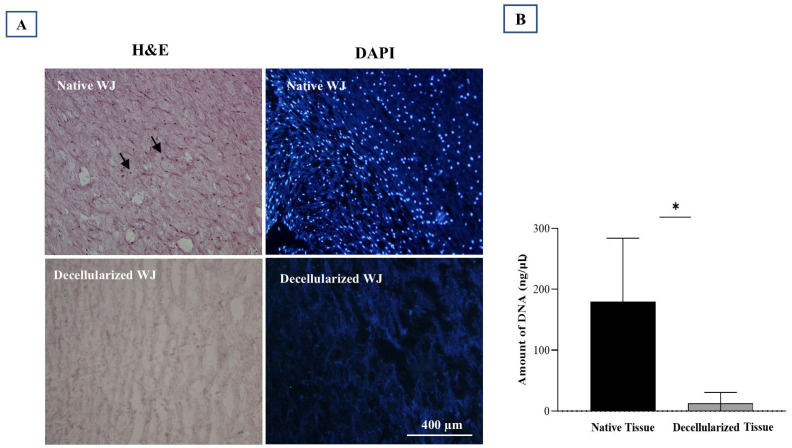
Evaluation of the decellularized Wharton’s Jelly (WJ). (**A**) Native vs. decellularized WJ comparison via H&E and DAPI staining. Black arrows (**left**) and shining blue dots (**right**) indicate cell nuclei in native tissue, absent in decellularized tissue. (**B**) DNA content assessment reveals a significant difference between native and decellularized tissues (* *p* value < 0.05). Three replications were performed for each assessment.

Figure 2E–G shows that decellularization eliminated the cells, as evidenced by the consistent white color of all samples after decellularization. To confirm the thorough removal of cellular components from the tissue, a combination of qualitative study (histological examination) and quantitative analysis (evaluation of DNA content) was conducted (Figure 3A,B).

Hematoxylin and eosin (H&E) evaluation demonstrated the effectiveness of our decellularization protocol in removing cells. It also successfully preserved the original three-dimensional structure of the extracellular matrix (ECM) in WJ tissue, which was similar to native tissue (Figure 3A). On the other hand, DAPI fluorescent staining showed that there were no visible cell nuclei, in contrast to native tissue (Figure 3A).

In addition, DNA content showed that the decellularized ECM had a significantly lower amount than the native tissue (*p* value = 0.030) (Figure 3B). 

### 3.2. Human Ovarian Follicle Isolation

**Figure 4:**
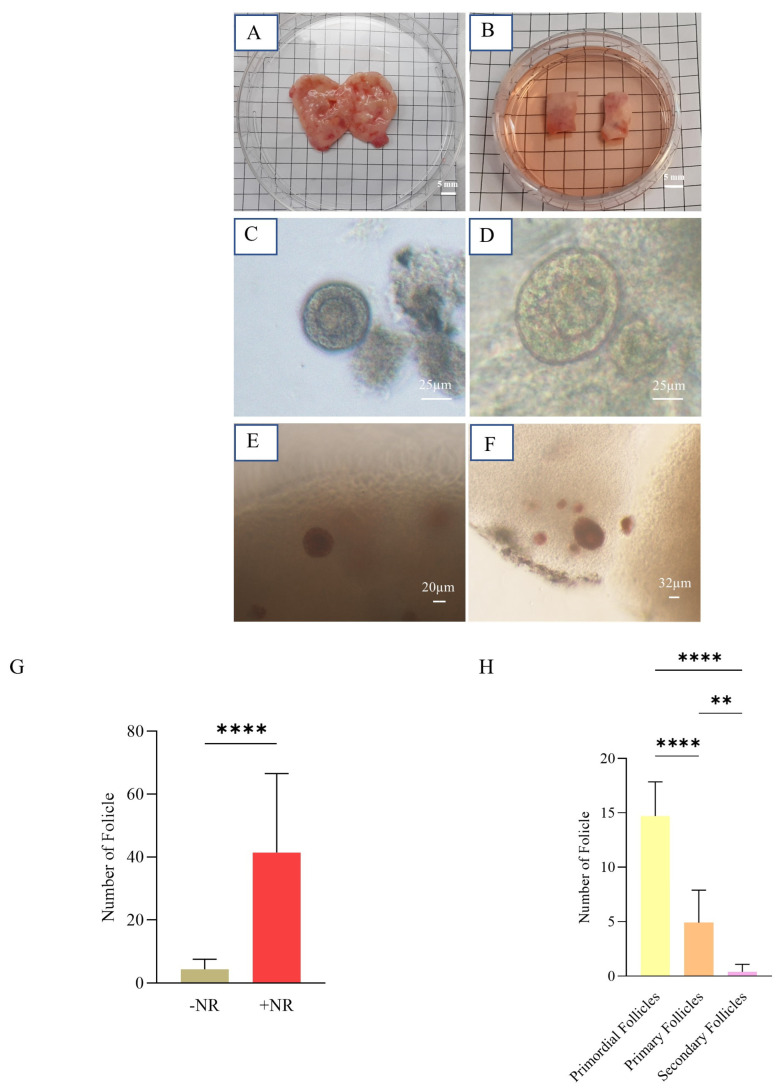
Isolation of human ovarian follicles. (**A**) An ovary from a transgender patient before removing its medulla. (**B**) Two vitrified-warmed strips (10 × 5 × 1 mm^3^). (**C**,**D**) Follicles without and (**E**,**F**) with NR dye. (**G**) Graph representing the mean number of partially isolated follicles with or without NR (+NR vs. −NR) (**** *p* value < 0.0001). (**H**) Graph representing the mean number of primordial, primary, and secondary partially isolated follicles in each of 20 follicles considered for an artificial ovary (** *p* value < 0.01; **** *p* value < 0.0001).

The comparison of follicles isolated with or without NR revealed a significant difference between the two groups. The number of isolated follicles was greater in the +NR group than in the −NR group (41.45 ± 25.14 vs. 4.36 ± 3.17), which can be attributed to the improved identification of follicles after staining (Figure 4G). This comparison shows an increase of 100% in the proportion of isolated follicles. Following isolation (as demonstrated in Figure 4H), approximately 160 follicles were retrieved from over 16 ovarian strips. Among them, 73.5% were classified as primordial follicles, which ranged between 30–60 µm. Primary follicles, measuring between 60 and 80 µm, made up 24.5% of the population. Secondary follicles, ranging between 80 and 150 µm, accounted for 2% of the population (Figure 4H).

**Figure 5:**
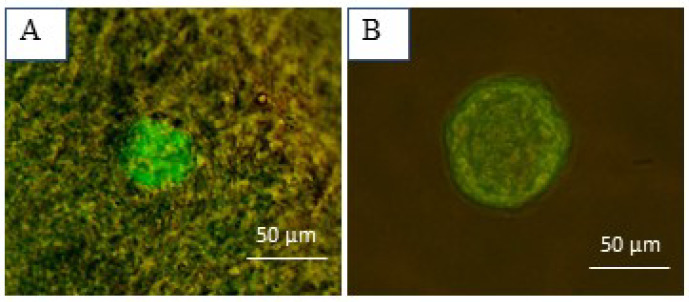
Follicular viability assessment using calcein-AM and ethidium homodimer-I. (**A**) A well-preserved primordial follicle embedded in the ovarian tissue and (**B**) an isolated secondary follicle.

To confirm follicle viability after isolation, around five to six random follicles per two strips were used for the live/dead assay (Figure 5). The results confirmed the viability of all these follicles.

### 3.3. Xenotransplanted Artificial Ovaries

**Figure 6:**
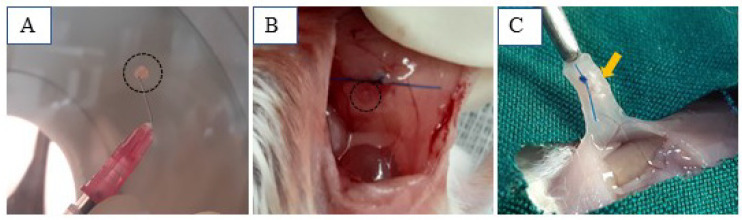
dWJ/Alg artificial ovaries in different conditions. (**A**) dWJ/Alg artificial ovary containing 20 human ovarian follicles before xenografting. (**B**,**C**) Transplantation site at the time of xenotransplantation (**B** (black circle) and 1 week later (**C** (yellow arrow)).

For every week (1, 2, 4, and 5), six artificial ovaries—three dWJ/Alg and three Alg—were grafted. Due to their distinct characteristics (as shown in Figure 6B), we were able to remove all of them successfully after the final timepoint (Figure 6C). Angiogenesis plays an important role in supporting the growth of transplanted, bioengineered ovaries and follicles. Narrow blood vessels can be observed at the graft site.

### 3.4. Histological Evaluation

**Figure 7:**
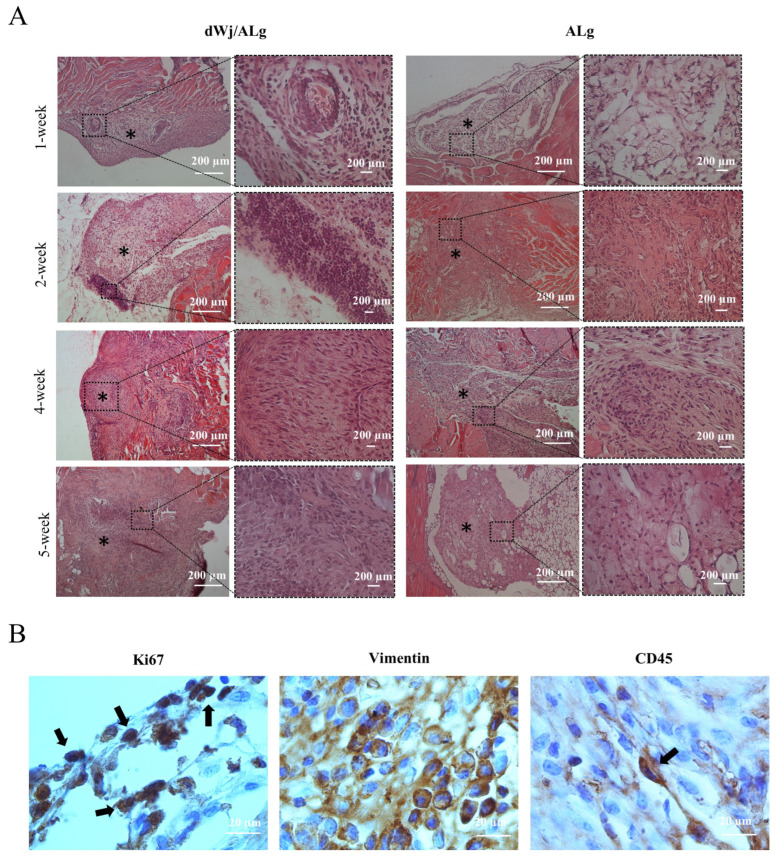
Histological analysis of transplanted artificial ovaries. (**A**) Histological staining was performed for dWJ/Alg and Alg artificial ovaries in different weeks (1, 2, 4, and 5 weeks). Although ovarian-like structures can be observed in all weeks, follicles can be seen in the 1-week xenotransplanted dWJ/Alg group.(Black stars (*) are ovarian-like structures) (**B**) Immunohistochemical staining. Vimentin- and Ki67-positive cells confirm the existence of human cells and their proliferation in the grafted dWJ/Alg artificial ovary (black arrows). Inflammatory CD45-positive cells can also be observed, but they are few in number (black arrow). Scale bar: 20 µm.

The histological assessment showed ovarian-like tissue in both groups 1 week post-xenografting. Figure 7A also displays the blood vessels detected in the histological sections. After one week, H&E evaluation of the xenotransplanted tissue revealed that the size of the follicles (52 ± 3.78 before xenotransplantation vs. 80.67 ± 12.42 after xenotransplantation) and the granulosa cell layer (changed to multilayer GC) in the dWJ/Alg group increased (Figure 4D). A survival rate of approximately 25% (in the primary follicle stage) was seen in the 1-week dWJ/Alg group. Meanwhile, the follicles and oocytes were eliminated in the Alg group and other weeks. The lack of angiogenesis and mouse immune system attacks may have a role in eliminating follicles in these groups. Furthermore, the histological assessment demonstrated the existence of ovarian-like structures with contained cell proliferation (Figure 7A). Conversely, in the dWJ/Alg group, five of twenty follicles reached the late secondary stage one week after xenotransplantation. In Figure 7A, the 1-week dWJ/Alg group increasing the GC layers is visible clearly. Additionally, the presence of fat-like structures can be seen in Figure 7 in 1 and 5 weeks of the Alg group. These sections may have been separated from the neighboring tissue, or, especially in week, 1 it may have appeared due to cell and tissue death. The appearance of fatty tissue sometimes results from the death of cells in the tissue.

### 3.5. Immunofluorescence Staining

Antibodies against Vimentin, Ki67, and CD45, respectively, identified the presence of human, proliferative, and inflammatory cells (Figure 7B). The high number of Vimentin-positive cells is evidence of the presence of human cells in the transplanted tissue. Proliferating cells were observed in all grafts starting in the initial week following transplantation. However, they were more numerous in the dWJ/Alg group (mean 16.6/120 m^2^ for dWJ/Alg and mean 9.2/120 m^2^ for Alg; *p* value = 0.01). Additionally, the Ki67 marker can be used to monitor proliferative cells and stain any GCs. The results of Ki67 staining indicate that the growth of GCs may have stopped after 1 week. However, this marker was seen in both groups (mean 21/120 m^2^ for dWJ/Alg and mean 8.2/120 m^2^ for Alg; *p* value = 0.07) On the other hand, some cells showed positive staining for mouse CD45. The CD45 marker was observed in greater abundance in the Alg group (mean 0.8/120 m^2^ for dWJ/Alg and mean 7/120 m^2^ for Alg; *p* value = 0.015). This means that some mouse inflammatory cells attacked the grafts (Figure 7B).

### 3.6. Hormonal Analysis

**Figure 8:**
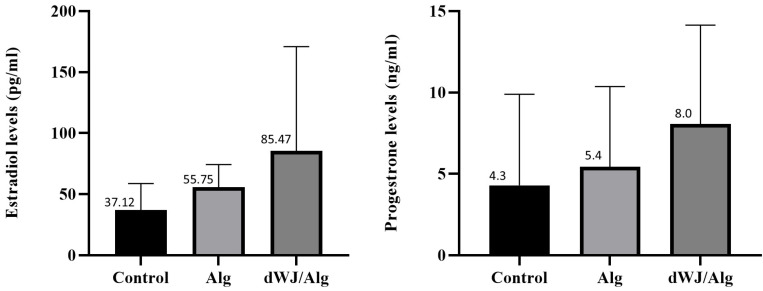
Hormonal assessments after artificial ovary xenotransplantation. Estradiol and progesterone level assessments showed that there was no significant difference in hormonal assay results among the WJ, Alg, and control groups after 1 week.

The hormonal analysis results for mouse blood serum are displayed in Figure 8. There was no significant difference in the level of either estradiol or progesterone between the dWJ/Alg and Alg groups.

## 4. Discussion

This study introduced a novel application of Wharton’s jelly, a well-known natural substance, for human artificial ovary construction (Figure 1). A Wharton’s jelly/alginate hydrogel combination was used for xenotransplantation, and alginate hydrogel was used in the control group. The findings indicate that Wharton’s jelly hydrogel-based artificial ovary effectively supports the viability and growth of isolated human follicles in xenotransplantation.

The number, developmental stages, and state of individual follicles within an artificial ovary can influence the subsequent outcomes [14]. The majority of the follicles in our trial were at the primordial stage. During this stage, the follicles typically reside close to one another. To minimize follicle loss or damage, they are commonly partially isolated to ensure all follicles are collected. This partial segregation allows the retrieval of additional follicles with reduced harm. Additionally, a vital dye called Neutral Red (NR) was used to identify specific follicles within the ovarian tissue. This aids us in accurately detecting and achieving a more thorough isolation of individual follicles. The most noteworthy observation was a significant increase in the number of isolated follicles after applying NR (Figure 4G,H).

The human ovarian tissues used in our investigation were collected from individuals who underwent hormone treatment as part of gender-affirming surgery (Figure 4A,B). These tissues were subsequently preserved in the Royan Human Ovarian Tissue Bank (Royan OTB) in Tehran, Iran, utilizing vitrification for research purposes. 

On the other side, hormone therapy in transgender patients may impact the contents of the follicles [39,40], suggesting that it may also influence the number of isolated follicles. The ovarian tissues used in the other investigations were taken from individuals undergoing laparoscopic surgery for benign gynecologic diseases [6,26,27,28,29].

In order to isolate healthy follicles, we employed a concentration of 1 mg/mL of collagenase IA. While the quantity of the enzyme can potentially affect the outcome of follicular isolation, our findings align with those of Mouloungui et al. They found no significant difference in the number or survival of follicles separated with collagenase NB6, Liberase DH, and collagenase IA [41]. Nevertheless, certain investigations indicate reduced rates of follicular isolation when utilizing liberases [26,27]. To prevent the recurrence of malignant cells, it is necessary to achieve complete follicle isolation at the clinical level.

Approximately ovarian-like structures could be seen in all groups (1 to 5 weeks after transplantation; Figure 7A). During transplantation, the artificial ovaries made by Alg cannot preserve the follicle structures. The results of the present study are consistent with the research conducted by Zand et al. [36] on mouse ovarian follicle autotransplantation using dWJ/Alg hydrogel and the comparison with the group that received Alg alone. Although two studies using mouse ovarian follicle transplantation [20,21] have demonstrated the success of alginate in encouraging follicle development, maturation to the MII stage, and embryo development, no human investigation has yet utilized alginate hydrogel. Concentration and cross-linking determine the stiffness of alginate, which can influence follicle survival, growth, and gene expression levels. However, the primordial follicles of primates require a more robust environment for growth, and the material properties of the scaffold should be adjusted accordingly [42]. This issue may limit the use of alginate in artificial ovary applications [12]. Various experiments [42] involved modifying the concentration of alginate, and further optimization may be necessary for human follicle culture.

Our findings indicate that the dWJ/Alg hydrogel effectively promotes the development of human ovarian follicles 1 week after grafting. Recently, there has been an increasing interest in utilizing decellularized extracellular matrix (ECM) in artificial ovary research. This is due to ECM’s biological activity and ability to signal growth factors and chemicals [43]. Following xenotransplantation, histological staining in the dWJ/Alg group, but not the Alg group, revealed an increase in the GC layers and follicle size (52 ± 3.78 before xenotransplantation vs. 80.67 ± 12.42 after xenotransplantation). As mentioned in the results, the 1-week dWJ/Alg group maintained approximately 25 percent of grafted follicles. A combination of growth factors, including bFGF, EGF, PDGF, and TGF-β, as well as the components of WJ, which include collagen, HA, and GAGs, may be presented [33,43] to promote follicle growth and improve scaffold bioactivity. This phenomenon is readily observable in histological evaluations. It is pleasing that our first human trial of the dWJ/Alg artificial ovary resulted in a 25% survival rate of follicles. In other human studies, the follicle survival rate ranges from 20% to 35%. It is worth noting that certain materials, such as plasma clots, have a history of resulting in offspring in mouse artificial ovary models [17,18].

In addition to ovarian-like structures, the presentation of Vimentin and Ki67 cells is hopeful. These findings demonstrate that human cells can survive and proliferate in dWJ/Alg AO. The quality of isolated follicles plays a key role in determining the successful transplantation [28]. While the live/dead assay verified the viability of isolated follicles, it is worth noting that most of the transplanted follicles in these experiments were preantral follicles [28,29]. Utilizing preantral follicles in advanced stages that have completed the activation phase appears to enhance this system. However, the 1-week dWJ/Alg group exhibited follicular growth.

Furthermore, there was no difference in hormone release between the transplanted and ovariectomized mice. Unfortunately, because of restrictions in our research, we have no access to nude or severe combined immunodeficient (SCID) mice. As a result, these disappointing data may be due to antigraft immune responses from immunocompetent mice and the destruction of the transplanted follicles.

## 5. Conclusions

The combination of Wharton’s jelly and alginate hydrogels has shown potential in supporting the human ovarian follicle graft and increasing follicle size. Therefore, this type of artificial ovary could be a beneficial option for supporting human ovarian follicles in short-term xenotransplantation. However, alginate cannot support the survival and growth of xenotransplanted follicles. On the other side, long-term xenotransplantation of dWJ/Alg had some limitations, including inadequate angiogenesis, delayed oxygen supply, and potential effects of the host immune system, which may impact the long-term results of xenotransplantation. Overcoming these limitations could pave the way for the use of Wharton’s jelly hydrogel as a functional artificial ovary at a later date.

## 6. Patents

This study was performed based on our previous research, registered patent number 98995 (international classification: A61L 27/52; C12N 5/00) at the State Organization for Registration of Deeds and Properties in Iran.

## Figures and Tables

**Table 1:** Decellularization process.

Steps	Materials	pH	Time (Hour)
1	10 mM Tris + 0.1% EDTA ^1^	8	16
2	10 mM base TBS ^2^ + 0.03% SDS ^3^ + 0.1% EDTA	7.6	24
3	Washing with Tris	-	-
4	50 mM Tris-HCl + 10 mM MgCl_2_	7.5	3
5	Washing with PBS	2 times, and each time 2 h	
	Washing with PBS + DNase/RNase	3 h	
	Washing with PBS	4–5 times, each time 2–3 h, and at least 2-overnight	

^1^ EDTA: ethylenediaminetetraacetic acid; ^2^ TBS: Tris-buffered saline; ^3^ SDS: sodium dodecyl sulfate.

## Data Availability

The original contributions presented in this study are included in the article. Further inquiries can be directed to the corresponding author(s).
